# Marine Natural Meroterpenes: Synthesis and Antiproliferative Activity

**DOI:** 10.3390/md8020347

**Published:** 2010-02-23

**Authors:** Annabel Simon-Levert, Christophe Menniti, Laurent Soulère, Anne-Marie Genevière, Chantal Barthomeuf, Bernard Banaigs, Anne Witczak

**Affiliations:** 1 Laboratoire de Chimie des Biomolécules et de l’Environnement EA4215, Université de Perpignan *via Domitia*, 52 avenue Paul Alduy, 66860 Perpignan Cedex, France; E-Mails: levert@univ-perp.fr (A.S.-L.); menniti@univ-perp.fr (C.M.); anne.witczak@univ-perp.fr (A.W.); 2 INSA Lyon, Laboratoire de Chimie Organique, Institut de Chimie et Biochimie Moléculaires et Supramoléculaires, Université de Lyon 1, UMR 5246, Bât J. Verne, 20 av A. Einstein, 69621 Villeurbanne Cedex, France; E-Mail: laurent.soulere@insa-lyon.fr; 3 Observatoire Océanologique de Banyuls Laboratoire Arago, CNRS-UMR7628/IPMC, 66650 Banyuls-sur-Mer, France; E-Mail: amg@obs-banyuls.fr; 4 Laboratoire de Pharmacognosie et Biotechnologies, Université d’Auvergne, 63001 Clermont-Ferrand, France; E-Mail: chantal.barthomeuf@u-clermont1.fr

**Keywords:** meroterpene, methoxyconidiol, epiconicol, didehydroconicol, antiproliferative activity

## Abstract

Meroterpenes are compounds of mixed biogenesis, isolated from plants, microorganisms and marine invertebrates. We have previously isolated and determined the structure for a series of meroterpenes extracted from the ascidian *Aplidium aff. densum*. Here, we demonstrate the chemical synthesis of three of them and their derivatives, and evaluate their biological activity on two bacterial strains, on sea urchin eggs, and on cancerous and healthy human cells.

## 1. Introduction

Meroterpenes are compounds of mixed biosynthesis, mostly quinone or hydroquinones bonded with a terpenoid portion ranging from one to nine isoprene units. In the marine environment, these secondary metabolites are isolated mainly from: brown algae such as *Cystoseira* [1], marine microorganisms [2], soft corals [3], or marine invertebrates, such as sponges or ascidians [4,5]. Some prenylated hydroquinones inhibit proliferation of a panel of cancer cells [6–8]. Moreover, three new sesqui- and diterpene hydroquinone MK2 or PI3 kinase inhibitors have been described from demosponges [9,10].

**Figure 1:**
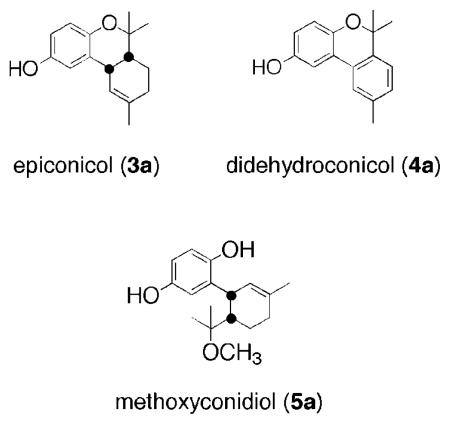
Structures of epiconicol (**3a**), didehydroconicol (**4a**) and methoxyconidiol (**5a**).

We recently extracted and elucidated the structure for a series of terpene hydroquinones from the ascidian *Aplidium aff. densum* [11]. Three of these secondary metabolites are shown in Figure 1.

One of these compounds, methoxyconidiol, when tested on sea urchin embryos during cell division, disturbs mitotic spindle assembly leading to a cell cycle arrest during metaphase/anaphase transition [12]. These promising results led us to synthesize compound **5a**, and the natural analogs **3a** and **4a**, as well as the methoxylated derivatives **3b**–**5b**, in order to determine the role of the phenol functions.

The antiproliferative activity of these products was tested on bacteria (*Micrococcus luteus* and *Escherichia coli*), sea urchin eggs and normal and cancerous human cells. Considering the severe cell cycle disruption produced by methoxyconidiol treatment in sea urchin eggs, we directly evaluated the cytotoxicity of these new compounds on a panel of murine and human cells, both healthy and cancerous.

## 2. Results and Discussion

### 2.1. Chemistry

**Scheme 1:**
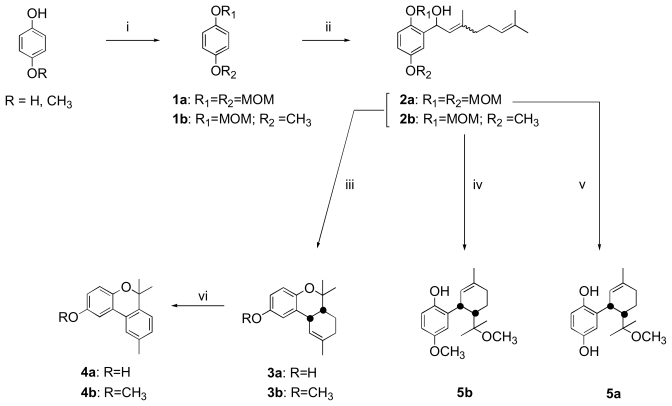
Reagents and conditions: (i) (a) NaH, THF, 1 h, rt, (b) MOMCl, THF, 3 h, rt; (ii) (a) nBuLi, THF, 2 h, rt, (b) citral, THF, 24 h, rt; (iii) HCl 6N, mixture 50: 50 THF/MeOH, 3 h, reflux; (iv) HCl 6N, MeOH, 3 h, rt; (v) HCl 12N, MeOH, 24 h, 8 °C; (vi) S, 4 h, 250 °C.

As previously described by Cruz-Almanza *et al.*, the natural meroterpene **3a** and the methoxy derivatives **3b**–**5b** were synthesized starting from compounds **2a** and **2b**, respectively, using acidic conditions (Scheme 1) [13]. These two geranylhydroquinone derivatives used as key intermediates were prepared from methoxymethyl (MOM) protected hydroquinone **1a** or MOM protected 4-methoxyphenol **1b** by aromatic lithiation and subsequent treatment with citral. The new natural meroterpene **5a** was synthesized by acidic treatment of **2a** in methanol for 24 hours at 8 °C. Reaction time (24h) and constant temperature (8 °C) were found to be crucial in obtaining this compound; other conditions (room temperature with a shorter reaction time) led to the meroterpene **3a**. The compound **5a** was thus synthesized with a yield of 25%. It should be noted that the acidic treatment of **2a** and **2b** promoted dehydration followed by a rearrangement of the corresponding carbocation leading to meroterpenes **3a**–**b** or **5a**–**b**. This reaction was found to be diastereoselective with a cis/trans ratio greater than 4/1. The pair of cis isomers was purified, and finally, good yields of the new natural chromene **4a** and its methoxy derivative **4b** were obtained (70% yields) from **3a** and **3b** by dehydrogenation in the presence of sulfur at 250 °C. The ^1^H and ^13^C NMR spectra of compounds **3a**, **4a** and **5a** were perfectly identical to those of the natural products epiconicol (**3a**), didehydroconicol (**4a**) and methoxyconidiol (**5a**) [11].

### 2.2. Biological activities

Some meroterpenes, of marine or plant origins, possess biological activities. Among them, cordiachromene A moderately inhibits the growth of *Micrococus luteus* [12], *Staphyclococcus aureus* and *Streptococcus faecalis* [14,15]. Hydroquinone sesquiterpenes are reported to be cytotoxic. Moreover, several meroterpenes isolated from terrestrial organisms inhibit the proliferation of a panel of cancer cells lines [9].

We evaluated the biological activities of compounds **3a**–**5a** and their methoxy derivatives **3b**–**5b**. Compounds **3b**–**5b** were designed and synthesized because the introduction of a methoxy group (instead of a phenolic group) could influence their biological activities. Antibacterial assays were carried out on two bacterial strains and antiproliferative assays were carried out against sea urchin eggs and a panel of cancerous and healthy cells.

#### 2.2.1. Antibacterial assays

The Microtiter Broth Dilution Method on gram negative (*Escherichia coli*) and gram positive (*Micrococcus luteus*) bacteria was used [16]. Meroterpenes dissolved in DMSO were added to bacteria suspensions (5:100 v:v) for 24 hours. The assay was carried out in triplicate. Bacterial growth was evaluated by absorbance measurement (630 nm). The minimum concentration that inhibits (MIC) bacterial growth was determined for each compound on each bacterial strain.

Four of the six meroterpenes had no effect on either of the two bacterial strains. Compounds **3a** and **4a** were weakly active. The MIC of epiconicol was 0.8 and 0.1 mM against *E. coli* and *M. luteus* respectively. Didehydroconicol inhibited the growth of *M. luteus* with a MIC of 0.5 mM. These two compounds possess phenolic group, and their substitution with a methoxy group (compounds **3b** and **4b**) led to totally inactive compounds.

#### 2.2.2. Antiproliferative activities

The six meroterpenes were further tested on two models: sea urchin eggs as well as on healthy and tumorous human cells. We first assayed the capacity of each compound to inhibit sea urchin egg divisions (*Paracentrotus lividus* and *Sphaerechinus granularis*). Early sea urchin embryos used to be a model organism for antiproliferative screening and identification of cellular targets. In fact, several physiological features make this system ideal for conducting a wide range of biological tests. These include straight forward artificial spawning, *in vitro* fertilization, rapid synchronous development, embryo optical transparency, and well understood embryogenesis.

For cytotoxic assays on sea urchins, meroterpenes were dissolved in an egg suspension (described in the experimental section), and their ability to inhibit 50% of cell division (IC_50_) was estimated. Each experiment was carried out in triplicate and an average taken. Results are shown in table 1.

Chromane (**3a)**, chromene (**4a**) and hydroquinone (**5a**) inhibit egg division, methoxyconidiol (**5a**) being the most active. IC_50_ of methoxyconidiol was 0.80 μM on *P. lividus* eggs and 4.30 μM on *S. granularis* eggs, while epiconicol (**3a**) inhibited *P. lividus* eggs division with a IC_50_ value of 9.80 μM and the IC_50_ of didehydroconicol (**4a**) was 11.30 μM on *P. lividus* eggs and was inactive on *S. granularis* eggs. Methoxylated analogs are not active on sea urchin eggs. The substitution of a phenolic function with a methoxy group in these compounds produced a total loss of activity.

To provide information on their antiproliferative activity against solid cancers, the six meroterpenes were tested on three human carcinoma cell lines corresponding to cancers commonly observed in Western countries: MCF7 (breast), PA1 (ovary) and PC3 (prostate). To obtain additional information on their specificity for human cell lines and their specificity for cancer *versus* normal cell lines, each compound was tested *versus* L-929 murine immortalized cells and human normal fibroblasts. To mimic what happens *in vivo*, assays were carried out on exponentially growing cells cultured in the presence of 10% serum. The growth inhibitory effect was determined by measuring, after a 48 hour exposure to each chemical, the metabolic activity of treated and control cultures using the resazurin reduction test (RRT), which measures the amount of fluorescent resofurin produced by living cells. The results clearly demonstrated the non toxicity in all cell lines of the three methoxylated analogs (**3b**–**5b**), didehydroconicol (**4a**) and methoxyconidiol (**5a**) with an IC_50_ > 100 μM. A dose-dependent inhibition of cell proliferation was only observed for epiconicol (**3a**). The IC_50_ of this product varied with the cell lines from 37 to 69 μM. The toxicity of epiconicol observed in our model is in agreement with data obtained by Caroll and co-workers on a panel of cancer cells [17].

**Figure 2:**
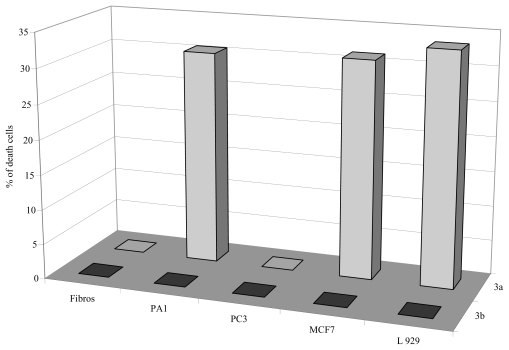
Effect of the compounds **3a** and **3b** on cell lines at 5 μM (PA1 ovarian, PC3 prostatical, MCF7 mammalian, human fibroblast and L929 murine fibroblasts). The antiproliferative effect was assessed by an evaluation of metabolical activity (RRT). Each experiment was carried out in triplicate.

At 5 μM, epiconicol showed a weak but significant selectivity for PA1 and MCF7 carcinoma cells. Accordingly, 30% of the PA1 and MCF7 cultures were killed, while any antiproliferative activity was observed on primary fibroblasts or androgen-resistant prostate carcinoma cells (PC3). Data in Figure 2 showed that for epiconicol, the replacement of the hydroxyl group with a methoxy group suppressed the cytotoxicity on PA1, MCF7 and L929 cells and, consequently the selectivity for these cell lines.

The susceptibility of sea urchin eggs and human cells to methoxyconidiol was compared. Methoxyconidiol has the capacity to inhibit the proliferation of sea urchin eggs but not that of human cancer cell lines. This may be due either to a problem of penetration through human cell membranes, or to the rapid detoxification of methoxyconidiol by human cells inactivating the compound, or to the rapid bioconversion of methoxyconidiol by sea urchin eggs activating the compound.

**Figure 3:**
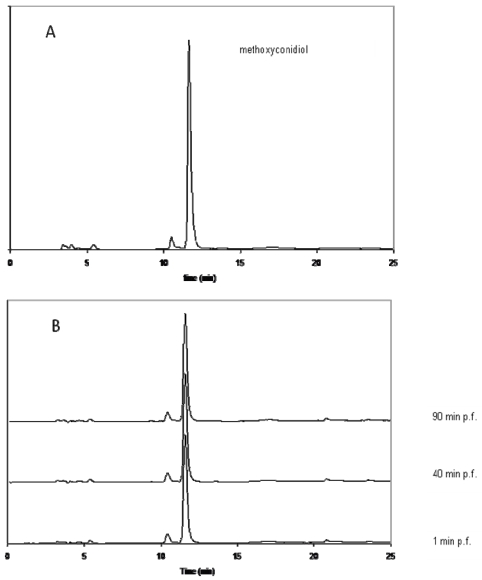
HPLC analysis of methoxyconidiol (System WATERS, column: synergy 4 μm RP max; elution water: methanol under a gradient; UV detection 285 nm; flow: 0.3 mL/min) A: control compound (methoxyconidiol) B: analysis after Solid Phase Extraction at 1, 40 and 90 minutes after fertilization (min p.f.).

To test the latter hypothesis, *S.s granularis* eggs were treated with 5 μM of methoxyconidiol, a concentration that inhibits their division. Sea urchin eggs were incubated 1, 40 and 90 minutes post-fertilization (p.f.) [18] in the presence of methoxyconidiol. Cytoplasmic extracts of treated and control eggs were further prepared. Organic extracts of the cytoplasmic preparations were finally obtained and analyzed by HPLC (Figure 3).

Chromatograms were compared to the one obtained with a pure methoxyconidiol solution. A single peak at a characteristic retention time and UV spectrum (photodiode array detection) for methoxyconidiol (**5a**) was observed in cytoplasmic extracts from sea urchin eggs. The level of the xenobiotic compound did not decrease significantly during incubation. No other minor compound appeared after 40 or 90 minutes of incubation suggesting that the antiproliferative compound on sea urchins is methoxyconidiol and not a biotransformed analog. Furthermore, the presence of methoxyconidiol on the cytoplasm clearly indicates that the compound penetrates through sea urchin cell membranes. Methoxyconidiol was found ineffective on human cancer cells: this can be explained by a difference in membrane permeability or intracellular transport of compounds between sea urchin cells and human cells, a phenomena previously described for some cells [19,20].

## 3. Experimental Section

### 3.1. Chemistry

Commercial reagents were used without further purification. Melting point was determined on a Köfler melting point apparatus and is uncorrected. ^1^H NMR (400 MHz) and ^13^C (100 MHz) were recorded on a JEOL EX 400 spectrometer. Chemical shifts are expressed in ppm from the solvent chloroform (CDCl_3_) used as an internal standard: δ_H_ 7.24 (residual CHCl_3_), δ_C_ 77.0 for CDCl_3_. Mass spectrum was recorded on an Automass Benchtop Quadripole Mass Spectrometer. Column chromatography was performed on silica gel 60 merck (0.063–0.200 mm). The meroterpenes **3a** and **3b**–**5b** were synthesized as previously described [13].

#### Procedures for the preparation of compounds 4a–5a and 4b

##### Methoxyconidiol (5a)

12 N hydrochloric acid (0.5 mL) was added dropwise to a stirred solution of alcohol **2a** (0.250 g, 0.7 mmol) in methanol (10 mL). The mixture was cooled at 8 °C for 24 h. The solution was diluted with a large excess of water and extracted with CH_2_Cl_2_. The combined organic layers were washed with water, dried (Na_2_SO_4_) and evaporated to produce a yellow oil, which was purified by the addition of a 8:2 mixture of ethyl acetate and heptane to furnish compound **5a** as a white solid (48 mg, 25%). Mp 197 °C. ^1^H NMR and ^13^C NMR, see reference 11; MS (EI) m/z: 276.

##### Didehydroconicol (4a)

A mixture of compound **3a** (0.154 g, 0.63 mmol) and sulfur (0.040 g, 1.26 mmol) was heated at 250 °C under a nitrogen atmosphere for 4h. The mixture was extracted with Et_2_O (2×10 mL). The solvent was evaporated and the residue was purified by column chromatography (heptane/ethyl acetate (8:2)) to furnish compound **4a** as a colorless oil (105 mg, 70%). ^1^H NMR and ^13^C NMR, see reference 11; MS (EI) m/z: 240.

##### *O*-methyldidehydroconicol (4b)

This compound was prepared from **3b** (0.140 g, 0.54 mmol) as described for **4a**. The residue was purified by column chromatography (heptane/ethyl acetate (95:5)) to give **4b** (96 mg) as a colorless oil with a yield of 70%. ^1^H NMR (CDCl_3_, 400 MHz): δ (ppm) 1.59 (s, 6H), 2.40 (s, 3H), 3.84 (s, 3H), 6.80 (dd, J = 9 Hz and J = 3 Hz), 6.87 (d, J = 9 Hz, 1H), 7.11–7.14 (m, 2H), 7.24 (d, J = 3 Hz, 1H), 7.49 (s, 1H). ^13^C NMR (CDCl_3_, 100 MHz): δ (ppm) 21.4, 27.5, 55.9, 77.3, 108.0, 115.0, 118.7, 122.9, 123.22, 123.20, 128.6, 128.9, 137.2, 137.3, 146.9, 154.4. MS (EI): m/z: 254.

### 3.2. Biological activities

The antibacterial assay used was based on the previously described method published by the National Committee of Laboratory Safety and Standards [11].

#### 3.2.1. Assays on sea urchin eggs

For antiproliferative assays on sea urchin eggs, we used a previously described method [12].

##### 3.2.1.1. Extraction of methoxyconidiol from treated sea urchin eggs

###### 3.2.1.1.1. Preparation of cytoplasmic extracts

Preparation of cytoplasmic extracts was based on the technique described by Collas and Poccia [18]. Gametes of *P. lividus* were collected in two separate beakers. Sperm was maintained undiluted on ice and eggs were stored at 19 °C until use. Sperm was diluted to 10% in Filtered Sea Water (FSW) and eggs were at 26 eggs/μL in FSW.

Fertilization was done in the proportion 1:1000 sperm:eggs (v:v). Elevation of the fertilization membrane was controlled under an inverted microscope (×10) and when 99% of the eggs were fertilized, methoxyconidiol dissolved in DMSO and FSW was added. A control without the addition of methoxyconidiol was carried out under the same conditions.

The cytoplasm of treated and control eggs was extracted just after fertilization, 40 and 90 minutes after fertilization. 10 mL of an homogeneous suspension of fertilized eggs was centrifuged at 2000 rpm for 5 min at 4 °C. 5 mL of Buffer A (10mM HEPES pH 8.0, 250 mM NaCl, 25 mM EGTA, 5 mM MgCl_2_, 110 mM glycine, 250 mM glycerol, 1 mM DTT and 1 mM PMSF) was added to the pellet and centrifuged again under the same conditions. Eggs were resuspended carefully in an ice-cold Buffer A at a volume ratio of 1:1 v:v and transferred into a 1.5 mL microcentrifuge tube on ice. To homogenize the solution, the eggs suspension was rapidly aspirated into a 3 mL syringe through a 22 gauge needle, expelled rapidly into the tube, re-aspirated and re-expelled quickly back into the tube. The lysate was centrifuged at 10,000 rpm for 10 min at 4 °C. An aliquot of the clear cytoplasm was taken, frozen in liquid nitrogen, and stored at −80 °C until chemical extraction.

###### 3.2.1.1.2. Chemical extraction of methoxyconidiol and HPLC analysis

Cytoplasmic extracts of treated and control eggs were extracted with CH_3_OH and CH_2_Cl_2_ and analyzed by HPLC. Small columns of reverse (C18) phase were prepared and conditioned with methanol, methanol:water 1:1 (v:v) and water. Cytoplasmic extracts were eluted from the column with water to eliminate polar compounds and salts. Methoxyconidiol was eluted with methanol and further concentrated. This fraction was analyzed in HPLC (column: Phenomenex synergy 4μ max RP; elution with water and methanol under gradient, starting from 30% to 0% of water during 15 minutes; flow: 0.3 mL/min; detection photodiode array, UV at 285 nm).

##### 3.2.1.2. Cell culture and cytotoxicity assay

Human primary fibroblasts were purchased from Biopredic International (Rennes, France). Immortalized murine L929 cells and human MCF7 breast adenocarcinoma, PA1 ovary teratocarcinoma and PC3 androgen-resistant prostate carcinoma cells were obtained from the European Collection of Cell Cultures (ECACC, Salibury, UK). All were cultured at 37 °C under 5% CO_2_ and maintained in Eagle’s minimum essential medium (Gibco-BRL, Paisley, Scotland) supplemented with 10% fetal calf serum, vitamins and antibiotics (Gibco^™^, Cergy-Pontoise, France) as described previously [21].

The cytotoxicity of each treatment was determined after a 48 h exposure to each chemical (6 concentrations in triplicate, each in DMSO 0.5%) by means of the resazurin reduction test (RRT) that measures the amount of fluorescent resorufin produced by living cells. Assays were carried out at 530/590 nm accordingly to the procedure described by Barthomeuf *et al.* [21]. IC_50_ values (mean ± S.D.) were calculated with the ALLFIT program and were expressed in μM.

## 4. Conclusions

Previous promising results [12] led us to synthesize six meroterpenes in order to evaluate their biological activities on two bacterial strains, on sea urchin eggs and on cancer cell lines. A first concern was to investigate the effect of a replacement of the phenolic hydroxyl by a methoxy group. In the present study, we conclude that this chemical modification reduced both the antibacterial and the antiproliferative activity of meroterpenes on sea urchin eggs and on normal, immortalized and cancer cell lines, highlighting the crucial role of the phenolic group at the C-4 position for the toxicity of meroterpenes. In contrast to previous findings on sea urchin eggs, methoxyconidiol was almost completely inactive on human serum-stimulated cells. We demonstrated that activity on sea urchin eggs was due to methoxyconidiol and not due to one of its degradation products. Furthermore, epiconicol, the most active compound on human cells, exhibited only medium toxicity on serumstimulated carcinoma cells. However, our data found evidence for a relative selectivity of this compound on serum-stimulated human MCF7 breast and PA1 ovary carcinoma cells.

## Figures and Tables

**Table 1:** Antiproliferative activity of meroterpenes on sea urchin eggs (*Paracentrotus lividus* and *Sphaerechinus granularis*). An egg suspension was incubated with the six compounds in a 96-well plate. 85 minutes after fertilization (time when 100% division occurred in the control eggs), the number of dividing eggs was recorded and IC_50_ was calculated for each compound. Each experiment was carried out in triplicate.

Compounds	IC_50_ (μM)	
	*P. lividus*	*S. granularis*
**3a**	>25.0	9.8 ± 1.3
**3b**	>25.0	>25.0
**4a**	11.3 ± 0.8	>25.0
**4b**	>25.0	>25.0
**5a**	4.3 ± 0.1	0.8 ± 0.0
**5b**	>25.0	>25.0
